# Evaluation of Hypofractionated Radiation Therapy Use and Patient-Reported Outcomes in Men With Nonmetastatic Prostate Cancer in Australia and New Zealand

**DOI:** 10.1001/jamanetworkopen.2021.29647

**Published:** 2021-11-01

**Authors:** David I. Pryor, Jarad M. Martin, Jeremy L. Millar, Heather Day, Wee Loon Ong, Marketa Skala, Liesel M. FitzGerald, Benjamin Hindson, Braden Higgs, Michael E. O’Callaghan, Farhan Syed, Amy J. Hayden, Sandra L. Turner, Nathan Papa

**Affiliations:** 1 Department of Radiation Oncology, Princess Alexandra Hospital, Brisbane, Queensland, Australia; 2 Australian Prostate Cancer Research Centre-QLD, Queensland University of Technology, Brisbane, Queensland, Australia; 3 Department of Radiation Oncology Calvary Mater Hospital Newcastle, Newcastle, New South Wales, Australia; 4 University of Newcastle School of Medicine and Public Health, Newcastle, New South Wales, Australia; 5 Monash University School of Public Health and Preventive Medicine, Melbourne, Victoria, Australia; 6 Alfred Health Radiation Oncology, Melbourne, Victoria, Australia; 7 Department of Radiation Oncology, Royal Hobart Hospital, Hobart, Tasmania, Australia; 8 Menzies Institute for Medical Research, University of Tasmania, Hobart, Tasmania, Australia; 9 Canterbury Regional Cancer and Haematology Service, Christchurch, New Zealand; 10 Department of Radiation Oncology, Royal Adelaide Hospital, Adelaide, South Australia, Australia; 11 University of South Australia, Adelaide, South Australia, Australia; 12 Urology Unit, Flinders Medical Centre, Bedford Park, South Australia, Australia; 13 Flinders Health and Medical Research Institute, Flinders University, Bedford Park, South Australia, Australia; 14 Department of Radiation Oncology, Canberra Hospital, Canberra, Australian Capital Territory, Australia; 15 ACRF Department of Cancer Biology and Therapeutics, John Curtin School of Medical Research, Australian National University, Canberra, Australian Capital Territory, Australia; 16 Sydney West Radiation Oncology, Westmead Hospital, Sydney, New South Wales, Australia; 17 Faculty of Medicine, Western Sydney University, Sydney, New South Wales, Australia; 18 Sydney Medical School, University of Sydney, Sydney, New South Wales, Australia

## Abstract

**Importance:**

Randomized clinical trials in prostate cancer have reported noninferior outcomes for hypofractionated radiation therapy (HRT) compared with conventional RT (CRT); however, uptake of HRT across jurisdictions is variable.

**Objective:**

To evaluate the use of HRT vs CRT in men with nonmetastatic prostate cancer and compare patient-reported outcomes (PROs) at a population level.

**Design, Setting, and Participants:**

Registry-based cohort study from the Australian and New Zealand Prostate Cancer Outcomes Registry (PCOR-ANZ). Participants were men with nonmetastatic prostate cancer treated with primary RT (excluding brachytherapy) from January 2016 to December 2019. Data were analyzed in March 2021.

**Exposures:**

HRT defined as 2.5 to 3.3 Gy and CRT defined as 1.7 to 2.3 Gy per fraction.

**Main Outcomes and Measures:**

Temporal trends and institutional, clinicopathological, and sociodemographic factors associated with use of HRT were analyzed. PROs were assessed 12 months following RT using the Expanded Prostate Cancer Index Composite (EPIC)–26 Short Form questionnaire. Differences in PROs were analyzed by adjusting for age and National Comprehensive Cancer Network risk category.

**Results:**

Of 8305 men identified as receiving primary RT, 6368 met the inclusion criteria for CRT (n = 4482) and HRT (n = 1886). The median age was 73.1 years (IQR, 68.2-77.3 years), 2.6% (168) had low risk, 45.7% (2911) had intermediate risk, 44.5% (2836) had high-/very high–risk, and 7.1% (453) had regional nodal disease. Use of HRT increased from 2.1% (9 of 435) in the first half of 2016 to 52.7% (539 of 1023) in the second half of 2019, with lower uptake in the high-/very high–risk (1.9% [4 of 215] to 42.4% [181 of 427]) compared with the intermediate-risk group (2.2% [4 of 185] to 67.6% [325 of 481]) (odds ratio, 0.26; 95% CI, 0.15-0.45). Substantial variability in the use of HRT for intermediate-risk disease remained at the institutional level (median 53.3%; range, 0%-100%) and clinician level (median 57.9%; range, 0%-100%) in the last 2 years of the study period. There were no clinically significant differences across EPIC-26 urinary and bowel functional domains or bother scores.

**Conclusions and Relevance:**

In this cohort study, use of HRT for prostate cancer increased substantially from 2016. This population-level data demonstrated clinically equivalent PROs and supports the continued implementation of HRT into routine practice. The wide variation in practice observed at the jurisdictional, institutional, and clinician level provides stakeholders with information that may be useful in targeting implementation strategies and benchmarking services.

## Key Points

**Question:**

What factors are associated with use of hypofractionated vs conventional radiation therapy for prostate cancer, and is there a difference in patient-reported outcomes (PROs) at a population level?

**Findings:**

In this cohort study of data from 6368 men with nonmetastatic prostate cancer, use of hypofractionation increased from 2.1% to 52.7% from the first half of 2016 to the second half of 2019 with no differences in PROs between those receiving hypofractionated radiation therapy and conventional radiation therapy. Substantial variation in use was found between jurisdictions, institutions, individual clinicians, and patient cohorts.

**Meaning:**

Findings of this cohort study support the continued implementation of hypofractionated radiation therapy into routine practice and provide stakeholders with information that may be useful in targeting implementation strategies.

## Teaser

This cohort study analyzes factors associated with the use of hypofractionated radiation therapy during the period from 2016 to 2019 and evaluates real-world patient-reported outcomes data for men with nonmetastatic prostate cancer receiving hypofractionated radiation therapy and conventional radiation therapy.

## Introduction

Level 1 evidence supports shorter fractionation schedules for men with prostate cancer receiving external beam radiation therapy (EBRT). In 2016 and early 2017, 3 large randomized clinical trials reported noninferiority of cancer control, toxic effects, and patient-reported outcomes (PROs) for short-course moderately hypofractionated EBRT (HRT) compared with long-course conventional radiation therapy (CRT).^1,2,3^ The 2018 American Society for Radiation Oncology, American Society of Clinical Oncology, and American Urological Association guideline update^4^ recommended that HRT be offered for any localized prostate cancer risk category when using EBRT and not treating the pelvic lymph nodes.

Systematic evaluations of how new evidence is implemented into real-world practice are often lacking. Uptake of HRT into routine practice has been shown to be highly variable across health care settings and at the individual clinician level.^5,6^ Uncertainty regarding the potential toxic effects of HRT remains a common concern regarding its adoption.^7^

The Australian and New Zealand Prostate Cancer Outcomes Registry (PCOR-ANZ) is a population-based clinical quality outcomes registry designed to monitor the incidence, patterns of care, and treatment outcomes for men with prostate cancer, including quality-of-life measures.^8,9,10^ The registry provides a mechanism to evaluate variations in patterns of care and outcomes and provides performance feedback to health care clinicians and hospital services via quality indicator reports and benchmarking. In Australia, each state manages and administers its own public hospital services, and approximately one-third of RT courses are delivered in the private sector. PCOR-ANZ encompasses registries in 8 jurisdictions and spans both public and private sectors, with population coverage of the registry reaching more than 70% in 2018. A major focus of PCOR-ANZ is the collection of PROs in the form of the Expanded Prostate Cancer Index Composite (EPIC)–26 Short Form questionnaire, currently collected at 12 months post treatment.^11^ This questionnaire allows for continued monitoring of PROs at a population level as patterns of care evolve.

In this first report to date of RT data from PCOR-ANZ, we aimed to analyze factors associated with the use of HRT during the period from 2016 to 2019 and evaluate real-world PRO data for men with nonmetastatic prostate cancer receiving HRT and CRT.

## Methods

PCOR-ANZ received ethical approval from the Monash University human research ethics committee, which does not require additional individual patient consent for analysis of registry data and is registered with the Australian Commission on Safety and Quality in Health Care register of clinical registries. This report follows the Strengthening the Reporting of Observational Studies in Epidemiology (STROBE) reporting guideline for cohort studies. Data from patients commencing primary treatment with EBRT from January 2016 to December 2019 were extracted from PCOR–ANZ in March 2021. Patients were excluded if they received additional brachytherapy, had metastatic disease at diagnosis, their National Comprehensive Cancer Network (NCCN) risk category was not recorded, or the number of fractions or total radiation dose was not recorded. We defined CRT as at least a 70-Gy cumulative dose in 1.7 to 2.3 Gy/fraction; HRT as a 54- to 70-Gy total dose in 2.5 to 3.3 Gy/fraction; and ultrahypofractionated RT (URT) as a 30- to 45-Gy dose in 5.5 to 8.5 Gy/fraction.^4^

Classification of socioeconomic status (SES) into quintiles based primarily on income and educational level attainment and location of residence into 3 categories (major city, inner regional, and outer regional/remote) was performed by obtaining postcode correspondence with the Australian Bureau of Statistics data sources^12,13^: Index of Relative Socioeconomic Advantage and Disadvantage 2016 and Australian Statistical Geography Standard: remoteness structure 2016. This information was not available for patients from New South Wales (NSW) or New Zealand.

Patient-reported quality of life was assessed by telephone, email, or post using the EPIC-26 questionnaire approximately 12 months following treatment.^11^ Responses to the EPIC-26 were transformed into summary scores (range, 0-100, with a higher score indicating a better function) for the domains of urinary incontinence, urinary irritation/obstruction, bowel function, and sexual function. Cronbach α was calculated for each domain. Responses to the specific urinary and bowel bother questions were transformed from a 5-point Likert scale into a no/small problem category and a moderate/big problem category to focus on those men experiencing a magnitude of problem that likely requires further assessment and intervention.

### Statistical Analysis

Temporal trends in the use of HRT vs CRT were examined over 6-month intervals by NCCN risk category and jurisdiction. Exact binomial CIs for proportion of HRT by year were calculated. Jurisdictions with small sample sizes were combined with a more populous sister state, Tasmania with Victoria, Australian Capital Territory (ACT) with NSW, and Northern Territory (NT) with South Australia (SA). Factors for HRT use included patient-level factors (age, NCCN risk category, SES, and residence), institution-level factors (public or private), and year of treatment. The associations between these variables and the outcome were assessed by simultaneously entering the variables into a multilevel mixed effects regression model with robust SEs and random intercepts for jurisdiction and institution and a random slope for private vs public status. A second multilevel mixed-effects model excluding these variables but using all patients was constructed using QR decomposition^14^ as an alternative estimation method. Patients with all covariate information were included in these models.

Variation in the uptake of HRT at the clinician level and institution level was graphically represented by funnel plots using the user-written command *funnelperform*. Differences between the schedules with respect to PROs were assessed with multivariable linear or logistic regression models after adjustment for age and NCCN risk category. Evaluation of the clinical significance of these differences was made with respect to estimates of clinically minimally important differences from the published literature: 4 to 6 for the bowel domain, 5 to 7 for the urinary irritative/obstructive domain, 6 to 9 for urinary incontinence, and 10 to 12 for the sexual domain.^15^ The summary domain scores were expressed as violin plots with the user-written command *vioplot*. Analyses were performed using Stata SE, version 14.0 (StataCorp LLC), with all tests 2-sided and significance set at *P* ≤ .05.

## Results

**Figure 1.**
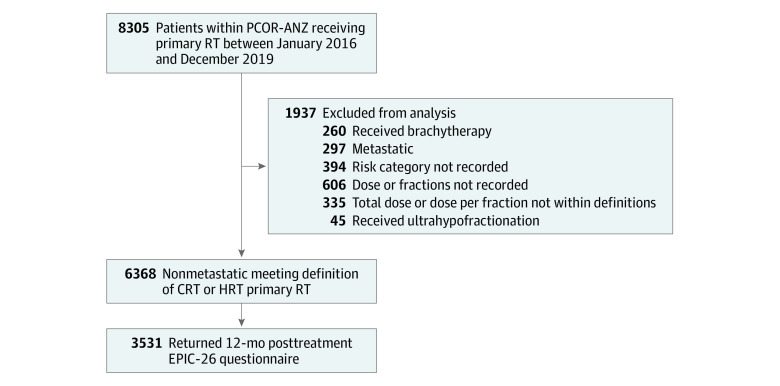
Patient Flow Diagram CRT indicates conventional radiation therapy; EPIC-26, Expanded Prostate Cancer Index Composite–26; HRT, hypofractionated radiation therapy; PCOR-ANZ, Australian and New Zealand Prostate Cancer Outcomes Registry; and RT, radiation therapy.

Within the 4-year period from 2016 to 2019, 8305 men had commenced RT for primary treatment of prostate cancer and consented to participate in PCOR-ANZ. Following application of the exclusion criteria, 6748 patients remained, 335 of whom had a recorded dose or dose/fraction not within the stated schedule definitions and 45 of whom received URT. The number of patients receiving URT was determined to be too small a sample for meaningful analysis, and those patients were excluded, leaving 6368 in the analysis (Figure 1).

Baseline characteristics are listed in Table 1. The median age at commencement of RT was 73.1 years (IQR, 68.2-77.3 years), with the most common NCCN risk categories being intermediate (45.7% [2911 of 6368]) and high/very high risk (44.5% [2836 of 6368]). Most patients were treated at a public institution (63.4% [4040 of 6368]) and lived in a major city (55.0% [2219 of 4035]).

**Table 1.** Characteristics of the Study Sample

Variable	No. (%)		
	Overall (N = 6368)	CRT (n = 4482)	HRT (n = 1886)
Age at RT commencement, median (IQR), y	73.1 (68.2-77.3)	73.2 (68.4-77.4)	72.6 (67.8-77.1)
Year of treatment			
2016	957 (15.0)	911 (20.3)	46 (2.4)
2017	1447 (22.7)	1168 (26.1)	279 (14.8)
2018	1834 (28.8)	1301 (29.0)	533 (28.3)
2019	2130 (33.4)	1102 (24.6)	1028 (54.5)
NCCN risk group			
Low	168 (2.6)	101 (2.3)	67 (3.6)
Intermediate	2911 (45.7)	1705 (38.0)	1206 (63.9)
High/very high	2836 (44.5)	2281 (50.9)	555 (29.4)
Regional (cN1)	453 (7.1)	395 (8.8)	58 (3.1)
Jurisdiction			
NSW/ACT	1717 (27.0)	1126 (25.1)	591 (31.3)
Victoria/Tasmania	1755 (27.6)	1361 (30.4)	394 (20.9)
Queensland	1506 (23.6)	1160 (25.9)	346 (18.3)
SA/NT	660 (10.4)	559 (12.5)	101 (5.4)
New Zealand	730 (11.5)	276 (6.2)	454 (24.1)
Institution			
Public	4040 (63.4)	2709 (60.4)	1331 (70.6)
Private	2309 (36.3)	1756 (39.2)	553 (29.3)
Not recorded	19 (0.3)	17 (0.4)	2 (0.1)
SES quintile^a^			
First (most disadvantaged)	876 (21.7)	689 (21.7)	187 (21.6)
Second	790 (19.6)	635 (20.0)	155 (17.9)
Third	827 (20.5)	652 (20.6)	175 (20.2)
Fourth	853 (21.1)	666 (21.0)	187 (21.6)
Fifth (most advantaged)	668 (16.6)	510 (16.1)	158 (18.2)
Not recorded	21 (0.5)	17 (0.5)	4 (0.5)
Location of residence^b^			
Major city	2219 (55.0)	1719 (54.2)	500 (57.7)
Inner regional	1069 (26.5)	819 (25.8)	250 (28.9)
Outer regional/remote	724 (17.9)	612 (19.3)	112 (12.9)
Not recorded	23 (0.6)	19 (0.6)	4 (0.5)

Abbreviations: cN1, regional nodal disease; CRT, conventional fractionated radiation therapy; HRT, moderately hypofractionated radiation therapy; NCCN, National Comprehensive Cancer Network; NSW/ACT, New South Wales and Australian Capital Territory; RT, radiation therapy; SA/NT, South Australia and Northern Territory; SES, socioeconomic status.

^a^ Data on SES quintile are not available for NSW and New Zealand. Column percentages exclude these jurisdictions. For overall, N = 4035; for CRT, n = 3169; and for HRT, n = 866.

^b^ Data on location of residence are not available for NSW and New Zealand. Column percentages exclude these jurisdictions. For overall, N = 4035; for CRT, n = 3169; and for HRT, n = 866.

**Figure 2.**
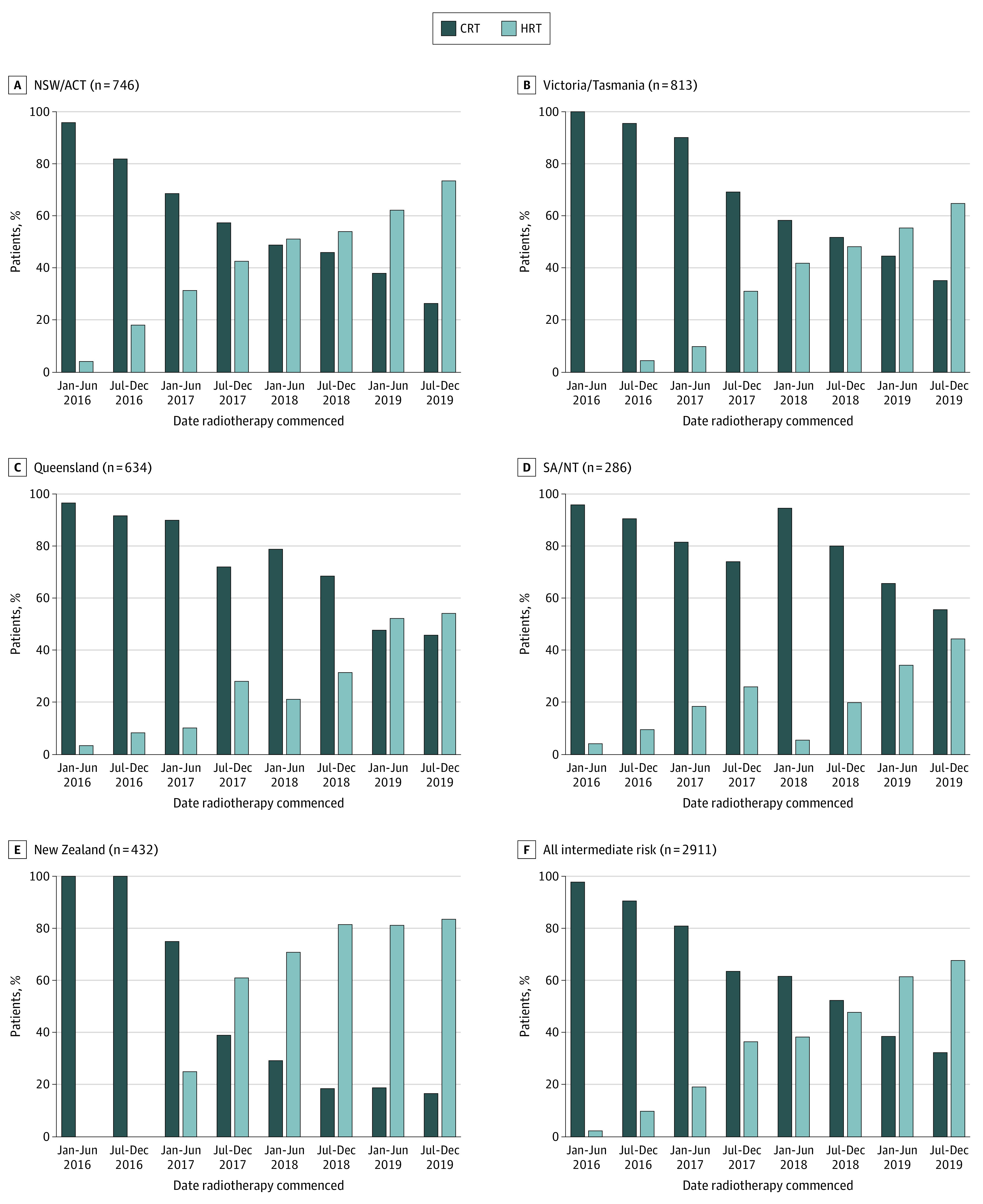
Variation in the Use of Moderately Hypofractionated Radiation Therapy (HRT) Over Time for Intermediate-Risk Prostate Cancer CRT indicates conventional radiation therapy; NSW/ACT, New South Wales and Australian Capital Territory; and SA/NT, South Australia and Northern Territory.

Overall, 4482 patients (70.4%) received CRT and 1886 patients (29.6%) received HRT. The use of HRT increased from 2.1% (9 of 435 patients) in the first half of 2016 to 52.7% (539 of 1023 patients) in the second half of 2019 (eFigure 1 and eTable 1 in the Supplement). This increase from the first half of 2016 to the second half of 2019 was especially pronounced for intermediate-risk cancer (2.2% [4 of 185] to 67.6% [325 of 481]) and was observed for high-/very high–risk disease (1.9% [4 of 215] to 42.4% [181 of 427]) and regional nodal disease (4.8% [1 of 21] to 23.1% [21 of 91]). Variation in the use of HRT over time for intermediate-risk cancer was observed between jurisdictions. In 2016 to 2017, the HRT proportion ranged from 13.1% (range, 9.8%-17.1%) in Victoria/Tasmania to 33.7% (range, 23.7%-44.9%) in New Zealand. In 2018 to 2019, the HRT proportion ranged from 22.0% (range, 15.9%-29.1%) in SA/NT to 80.5% (range, 76.0%-84.5%) in New Zealand (Figure 2; eTable 2 in the Supplement). Rapid adoption was apparent from 2017 for New Zealand whereas year-on-year increases in HRT proportions of approximately 15% were seen in NSW/ACT and Victoria/Tasmania with lower proportions in Queensland and SA/NT. Institutions that were part of the registry since 2016 contributed 86.9% (5524 of 6356) of patients and had a smaller proportion of HRT use in the second half of 2019 (48.9% [383 of 784] vs 65.3% [154 of 236]). Wide variation was noted in the use of HRT for intermediate-risk disease in the years 2018-2019 at the institutional level (median 53.3%; range, 0%-100%) and clinician level (median 57.9%; range, 0%-100%) (eFigure 2 in the Supplement). For institutions that treated at least 5 patients at intermediate risk for 2018 to 2019 (n = 65), 8 used HRT more than 95% of the time and 5 institutions used this schedule in less than 5% of their patients.

In the regression model that did not include SES quintile and location of residence as covariates (model 2), age, year of treatment, and NCCN risk group were strong predictors of receiving HRT (Table 2). In the regression model that did include SES quintile and location of residence but excluded patients from NSW and New Zealand (model 1), age, year of treatment, and NCCN risk group were also independently associated with receiving HRT. Multivariable analysis demonstrated that patient factors (such as increasing age) were also independently associated with receipt of HRT within PCOR-ANZ. Age per 5-year increase was associated with a 22% increase in odds of receiving HRT (odds ratio [OR], 1.22; 95% CI, 1.18–1.26). Men commencing RT in 2018 had more than 3 times the odds of receiving HRT (OR, 3.04; 95% CI, 1.84-5.00) than those commencing RT in 2016, and those commencing RT in 2019 had more than 7 times the odds of receiving HRT in 2017 (OR, 7.18; 95% CI, 5.08-10.16) than those commencing HRT in 2016. Conversely, patients in the high-/very high–risk category had 74% lower odds of receiving HRT (OR, 0.26; 95% CI, 0.15-0.45) than patients at intermediate risk. HRT use was higher in patients treated in a public institution and from a major city, but these findings were not statistically significant.

**Table 2.** Regression for Factors Associated With Receiving Moderately Hypofractionated Radiation Therapy

Variable	OR (95% CI)	
	Model 1	Model 2
Age at RT per 5-y increase	1.22 (1.18-1.26)	1.15 (1.09-1.21)
Year of treatment		
2016/2017	1 [Reference]	1 [Reference]
2018	3.04 (1.84-5.00)	3.23 (2.69-3.89)
2019	7.18 (5.08-10.16)	8.43 (7.00-10.14)
NCCN risk group		
Low	1.07 (0.84-1.36)	1.31 (0.88-1.95)
Intermediate	1 [Reference]	1 [Reference]
High/very high	0.26 (0.15-0.45)	0.22 (0.19-0.26)
Regional (cN1)	0.19 (0.13-0.27)	0.10 (0.08-0.15)
Institution		
Public	1 [Reference]	1 [Reference]
Private	0.92 (0.57-1.47)	0.66 (0.33-1.30)
SES quintile^a^		
First (most disadvantaged)	1 [Reference]	NA
Second	0.95 (0.76-1.19)	
Third	0.82 (0.71-0.94)	
Fourth	0.85 (0.58-1.26)	
Fifth (most advantaged)	0.88 (0.69-1.13)	
Location of residence^a^		
Major city	1 [Reference]	NA
Inner regional	0.99 (0.77-1.29)	
Outer regional/remote	0.63 (0.21-1.88)	

Abbreviations: cN1, regional nodal disease; OR, odds ratio; NA, not applicable; NCCN, National Comprehensive Cancer Network; RT, radiation therapy; SES, socioeconomic status.

^a^ Data on SES quintile and location of residences are not available for New South Wales and New Zealand. Model 1 incorporates these variables but excludes these patients (n = 3992). Model 2 uses the entire sample but does not include SES and location of residence as covariates (n = 6344).

The median time after RT commencement to completion of the EPIC-26 survey was 407 days (IQR, 371-449 days). EPIC-26 questionnaires were completed by 3531 patients (55.4%). Similar distributions in quality-of-life domain summary scores between the regimens were observed (eFigure 3 in the Supplement). The instrument demonstrated reasonable internal consistency (Cronbach α = 0.64-0.88) (eTable 3 in the Supplement). Following adjustment for age and risk category, the mean domain score for bowel function was 1.70 points higher (95% CI, 0.40-3.05 points) in HRT recipients; for the urinary irritation/obstruction domain, 1.21 points higher (95% CI, 0.00-2.43 points); and in the sexual function domain, 3.32 points higher (95% CI, 1.52-5.12 points) (Table 3). Although these findings were statistically significant, the adjusted mean differences were smaller than the published estimates of clinically minimally important differences.^15^ No statistically significant differences were noted in the urinary incontinence domain or dichotomized bother scores between regimens. The percentage of patients reporting moderate/big problem urinary bother was 9.8% (104 of 1058) for HRT vs 11.2% (272 of 2420) for CRT (adjusted OR, 0.96; 95% CI, 0.75-1.23; *P* = .80) and bowel bother was 8.3% (88 of 1060) for HRT vs 10.6% (258 of 2431) for CRT (adjusted OR, 0.83; 95% CI, 0.63-1.08; *P* = .16).

**Table 3.** EPIC-26 Domain Scores^a^

Domain and measurement	No. (%)	
	CRT	HRT
**Urinary incontinence domain**		
No.	2347	1023
Median (IQR)	100 (79 to 100)	100 (79 to 100)
Mean (SD)	86.1 (20.0)	87.5 (18.4)
Adjusted mean difference (95% CI) compared with CRT	NA	0.62 (−0.87 to 2.11)
**Urinary irritation/obstruction domain**		
No.	2285	993
Median (IQR)	88 (75 to 100)	94 (81 to 100)
Mean (SD)	85.6 (15.9)	87.0 (15.2)
Adjusted mean difference (95% CI)	NA	1.21 (0.00 to 2.43)
**Bowel function domain**		
No.	2333	1013
Median (IQR)	92 (79 to 100)	96 (83 to 100)
Mean (SD)	86.2 (18.3)	88.1 (16.1)
Adjusted mean difference (95% CI)	NA	1.70 (0.40 to 3.05)
**Sexual function domain**		
No.	2237	1014
Median (IQR)	17 (6 to 26)	18 (10 to 45)
Mean (SD)	22.5 (23.6)	30.1 (27.4)
Adjusted mean difference (95% CI)	NA	3.32 (1.52 to 5.12)
**Urinary bother**		
No.	2420	1058
No/small problem	2148 (88.8)	954 (90.2)
Moderate/big problem	272 (11.2)	104 (9.8)
Adjusted odds ratio (95% CI)	[Reference]	0.96 (0.75 to 1.23)
**Bowel bother**		
No.	2431	1060
No/small problem	2173 (89.4)	972 (91.7)
Moderate/big problem	258 (10.6)	88 (8.3)
Adjusted odds ratio (95% CI)	[Reference]	0.83 (0.63 to 1.08)

Abbreviations: CRT, conventional fractionated radiation therapy; EPIC-26, Expanded Prostate Cancer Index Composite-26; HRT, moderately hypofractionated radiation therapy; NA, not applicable.

^a^ The different sample sizes are owing to the fact that not all questions in the EPIC-26 survey were answered by all men.

## Discussion

This cohort study, the first evaluation to date of radiation therapy data from PCOR-ANZ, provides insight into the variable implementation of HRT in a real-world setting. The finding of similar PROs between CRT use and HRT use at a population level is consistent with the randomized clinical trials and supports the continued implementation of HRT into routine practice. While a substantial increase in use of HRT was observed in the years following publication of the seminal noninferiority studies in 2016, the registry data highlighted substantial variation in HRT use across jurisdictions, institutions, clinicians, and patient cohorts. These data may be used by stakeholders and jurisdictions to help inform implementation strategies and benchmarking of their services.

An increase in use was most evident in the intermediate-risk group, increasing from 2% in the first half of 2016 to 68% by 2019. In later years, a more modest increase in use of HRT for high-/very high–risk disease was observed (up to 42%), consistent with evolving guidelines in 2018 recommending HRT regardless of risk group, when not including elective pelvic nodes.^4^ The use of elective pelvic nodal fields was not collected as part of the minimum data set. A smaller increase in use of HRT was seen in the regional nodal group up to 23% in the second half of 2019. The lower use of HRT in patients with node positivity is consistent with American Society for Radiation Oncology guidelines over the study period specifically excluding pelvic lymph node radiotherapy from HRT recommendations. The recent NRG Oncology consensus atlas^16^ on pelvic lymph node volumes for prostate cancer RT incorporates suggested dose constraints for commonly used HRT schedules. The low-risk subgroup comprised only 2.6% of the cohort treated with definitive EBRT, consistent with the increasing use of active surveillance.^17^ The use of URT or stereotactic body radiation therapy (SBRT) in PCOR-ANZ during the study period was low (<1%) but may be expected to increase in subsequent years.

A major strength of PCOR-ANZ is the routine collection of PROs that can be monitored at a population level as patterns of care evolve. Within the current analysis, a subset of 55% of men returned 12-month posttreatment EPIC-26 questionnaires. Receipt of HRT was associated with marginally higher scores in the urinary irritation/obstruction, bowel function, and sexual function domains (mean adjusted differences of 1.21 points for urinary, 1.70 points for bowel function, and 3.32 points for sexual function); findings were statistically significant but lower than published estimates of clinically relevant minimally important difference thresholds.^15^ No significant differences were seen in the urinary incontinence domain or in the urinary and bowel bother scores. These real-world results are in keeping with the Conventional or Hypofractionated High Dose Intensity Modulated Radiotherapy in Prostate Cancer (CHHiP) and Radiation Therapy Oncology Group 0415 studies that found no clinically meaningful differences in PROs between HRT and CRT.^18,19^ The proportion of men reporting moderate or big urinary or bowel bother was less than 10% and was consistent with the CHHip study.^20^ A national cohort study from England^18^ also reported no differences in EPIC-26 urinary and bowel function between men receiving HRT or CRT, with mean scores in the order of 86 across the domains and consistent with the findings in this cohort study.

Variable patterns of uptake have been reported in different health care settings.^5,20,21^ PCOR-ANZ is a binational collaboration between Australia and New Zealand. While each country’s health care system shares similarities (access to universal health care being key), funding models and the proportion of RT delivered by the private sector differ (36% in Australia vs 10% in New Zealand).^22^ Funding in Australia for both private and public sectors retains a component of activity-based and fee-for-service models proportionate to the number of fractions delivered, whereas public funding in New Zealand provides a global budget for all services by each facility. By the second half of 2019, the proportion of men receiving HRT in New Zealand was 73% compared with 35% to 58% among Australian jurisdictions.

In the UK, where a universal health care model is delivered by the National Health Service (NHS), the use of HRT increased from 8% in 2012-2013 to 49% in 2016-2017 following a conference report of initial results of the CHHiP trial later published.^20^ In 2017, NHS England undertook to audit RT practice against a benchmark usage rate of 70% for HRT. By 2019, the proportion of men with intermediate-risk disease receiving HRT had increased to 96%.^23^

In the US, analyses of the National Cancer Database have reported significant increases in the use of SBRT from less than 1% in 2004 to 7% to 10% by 2015, while use of HRT remained relatively unchanged at 6%.^21,24^ Socioeconomic and geographic disparities in the US have been noted, with use of SBRT associated with treatment at academic centers, living in an urban area, higher income, White race, and fewer comorbidities.^24^ The use of HRT and SBRT will likely increase further and be implemented more broadly with the continuing evolution of the HRT evidence base, moves toward value-based payment models, and the current COVID-19 pandemic.^25,26,27,28,29^

In addition to NCCN risk grouping, multivariable analysis suggested that patient factors (such as increasing age) were also independently associated with receipt of HRT within PCOR-ANZ, perhaps reflecting residual concerns among clinicians regarding potential late effects with HRT schedules.^7^ The funnel plots (eFigure 2 in the Supplement) highlight the wide variation in use at an individual institution and clinician level for the 2018-2019 period. These findings are similar to a 2016 report by Delaney and colleagues^6^ examining factors associated with use of HRT for breast cancer in Australia, suggesting a need for both systemwide and individual concerns to be analyzed and addressed.

A subset of jurisdictions contributed data on geographic area of residence, allowing for analysis of derived socioeconomic indexes. While no statistically significant results were found between the private and public sector, location of residence or SES quintiles, usage rates of HRT in regional and rural areas, private institutions, and more advantaged SES quintiles warrant closer analysis and an investment in harmonization of data collection across jurisdictions as the registry matures.

The aim of clinical quality registries such as PCOR-ANZ is to monitor patterns of care, outcomes, and variance as well as to use this information to reduce variations and disparities in care and improve outcomes. Our analysis has highlighted the importance of monitoring the implementation of evidence-based care into practice and provides further insight into the multiple potential factors behind this variation that may need to be addressed to optimize implementation strategies. These real-world data also provide confirmation that high-quality PROs are being maintained as hypofractionation use increases across Australia and New Zealand.

A metric of hypofractionation use will be incorporated into future PCOR-ANZ biannual reports sent to participating clinicians and institutions to facilitate feedback and audit and benchmarking efforts. A metric of hypofractionation may also provide more timely feedback in response to the changing evidence base (for example, URT and SBRT) or strains on the health care system, such as the COVID-19 pandemic.

### Limitations

This study has limitations. First, PCOR-ANZ continues to expand as the number of health services reporting to the registry increases. Population coverage increased from 40% in 2016 to more than 70% in 2018. Bringing new health services into the registry may have contributed to some of the observed changes in HRT use. Furthermore, this analysis covered the period from 2016 to 2019 prior to the COVID-19 pandemic. Although Australia and New Zealand largely avoided broadscale community transmission, the pandemic will likely have transformative and long-lasting consequences for our health care systems and may be a further catalyst for accelerating the adoption of evidence-based hypofractionation and SBRT for prostate cancer, which can be monitored in the coming years.^29^ The inclusion of pretreatment PROs and comorbidity indexes may have enhanced these analyses but were not collected within the registry data set. In addition, to our knowledge, this cohort study was the first detailed analysis of RT data within PCOR-ANZ. Although approximately 20% of potentially eligible patients were excluded because of missing staging data (5%), missing dose or fractionation data (10%), or dose/fractionation schemes not within the definition (5%), initiatives are under way to help improve data completeness and interfaces with existing radiation oncology data repositories.

## Conclusions

In this cohort study, usage rates of HRT for prostate cancer increased substantially in Australia and New Zealand since 2016. Our population-level data reporting equivalent PROs with HRT and CRT are consistent with randomized clinical trials and support the continued implementation of HRT into routine practice. The wide variation in practice at the jurisdictional, institutional, and clinician levels provides stakeholders with information that may be useful in targeting implementation strategies and benchmarking their services.

## Notes

Supplement. **eFigure 1.** Proportion of All Patients Receiving CRT Versus HRT per Six-Month Interval, Overall and by NCCN Risk Group **eFigure 2.** Funnel Plots by Clinician and Institution of Case Volume Versus Proportion HRT Treatment for Intermediate Risk Prostate Cancer in the Years 2018-19. Red line = mean, dashed line = one standard deviation. Minimum five patients per clinician or institution **eFigure 3.** Violin Plot of EPIC-26 Domain Scores for Urinary Incontinence, Urinary Irritation/Obstruction, Bowel Function, and Sexual Function by Radiation Therapy Schedule **eTable 1.** Percentage Moderately Hypofractionated Radiation Therapy and 95% Exact Binomial Confidence Interval (CI) by Year and NCCN Risk Category **eTable 2.** Percentage Moderately Hypofractionated Radiation Therapy and 95% Exact Binomial Confidence Interval (CI) by Year and Jurisdiction for NCCN Intermediate Risk Disease **eTable 3.** Characteristics of Domain Specific Scores of EPIC-26 in the Sample
