# Supplementary material for: Evaluation of Hypofractionated Radiation Therapy Use and Patient-Reported Outcomes in Men With Nonmetastatic Prostate Cancer in Australia and New Zealand
Source: JAMA Netw Open. 2021 Nov 1;4(11):e2129647. doi: 10.1001/jamanetworkopen.2021.29647 (PMC8561328; doi:10.1001/jamanetworkopen.2021.29647)
Supplement: Supplement. — eFigure 1. Proportion of All Patients Receiving CRT Versus HRT per Six-Month Interval, Overall and by NCCN Risk Group eFigure 2. Funnel Plots by Clinician and Institution of Case Volume Versus Proportion HRT Treatment for Intermediate Risk Prostate Cancer in the Years 2018-19. Red line = mean, dashed line = one standard deviation. Minimum five patients per clinician or institution eFigure 3. Violin Plot of EPIC-26 Domain Scores for Urinary Incontinence, Urinary Irritation/Obstruction, Bowel Function, and Sexual Function by Radiation Therapy Schedule eTable 1. Percentage Moderately Hypofractionated Radiation Therapy and 95% Exact Binomial Confidence Interval (CI) by Year and NCCN Risk Category eTable 2. Percentage Moderately Hypofractionated Radiation Therapy and 95% Exact Binomial Confidence Interval (CI) by Year and Jurisdiction for NCCN Intermediate Risk Disease eTable 3. Characteristics of Domain Specific Scores of EPIC-26 in the Sample [file jamanetwopen-e2129647-s001.pdf]

Supplemental Online Content

Pryor DI, Martin JM, Millar JL, et al. Evaluation of hypofractionated radiation therapy use and patient-reported outcomes in men with nonmetastatic prostate cancer in Australia and New Zealand. *JAMA Netw Open*. 2021;4(11):e2129647. doi:10.1001/jamanetworkopen.2021.29647

eFigure 1. Proportion of All Patients Receiving CRT Versus HRT per Six-month Interval, Overall and by NCCN Risk Group
eFigure 2. Funnel Plots by Clinician and Institution of Case Volume Versus Proportion HRT Treatment for Intermediate Risk Prostate Cancer in the Years 2018-19. Red Line = Mean, Dashed Line = One Standard Deviation. Minimum Five Patients per Clinician or Institution
eFigure 3. Violin Plot of EPIC-26 Domain Scores for Urinary Incontinence, Urinary Irritation/obstruction, Bowel Function, and Sexual Function by Radiation Therapy Schedule
eTable 1. Percentage Moderately Hypofractionated Radiation Therapy and 95% Exact Binomial Confidence Interval (CI) by Year and NCCN Risk Category
eTable 2. Percentage Moderately Hypofractionated Radiation Therapy and 95% Exact Binomial Confidence Interval (CI) by Year and Jurisdiction for NCCN Intermediate Risk Disease
eTable 3. Characteristics of Domain Specific Scores of EPIC-26 in the Sample

This supplemental material has been provided by the authors to give readers additional information about their work.

© 2021 Pryor DI et al. *JAMA Network Open*.

eFigure 1. Proportion of all patients receiving CRT versus HRT per six-month interval, overall and by NCCN risk group.

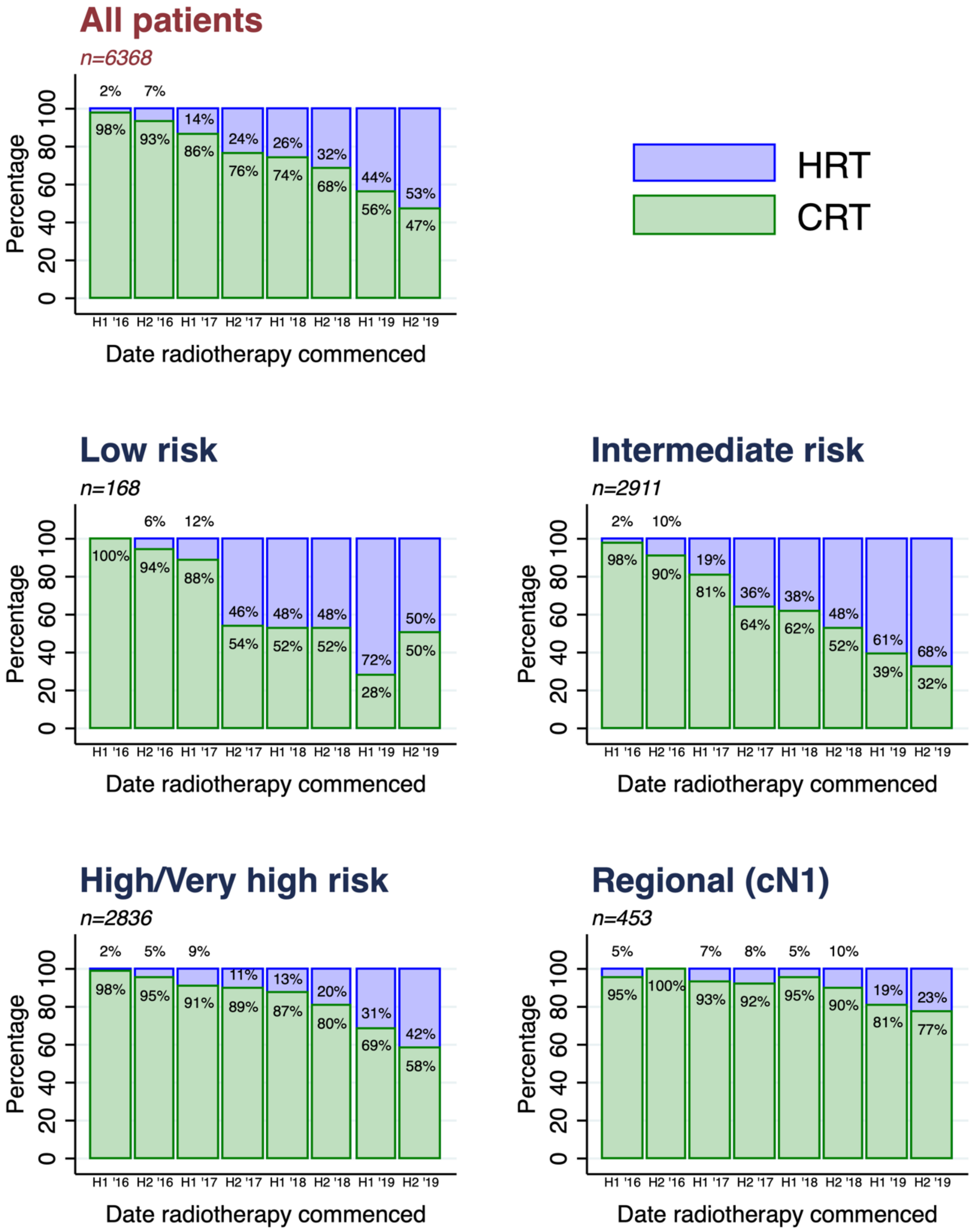

© 2021 Pryor DI et al. *JAMA Network Open*.

eFigure 2. Funnel plots by clinician and institution of case volume versus proportion HRT treatment for intermediate risk prostate cancer in the years 2018-19. Red line = mean, dashed line = one standard deviation. Minimum five patients per clinician or institution.

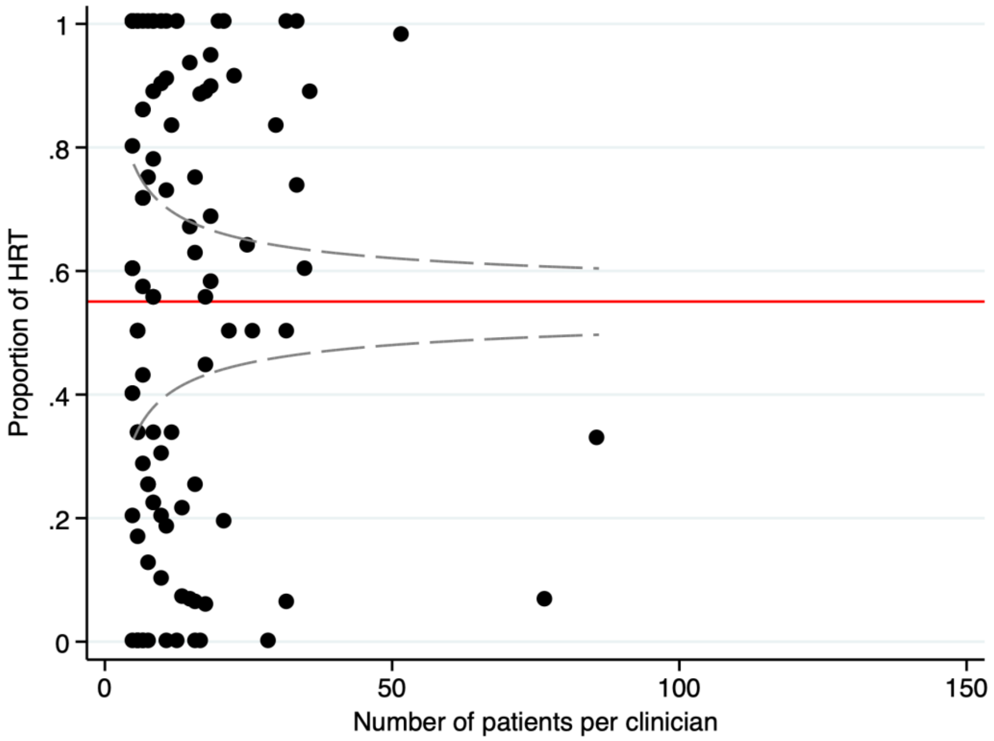

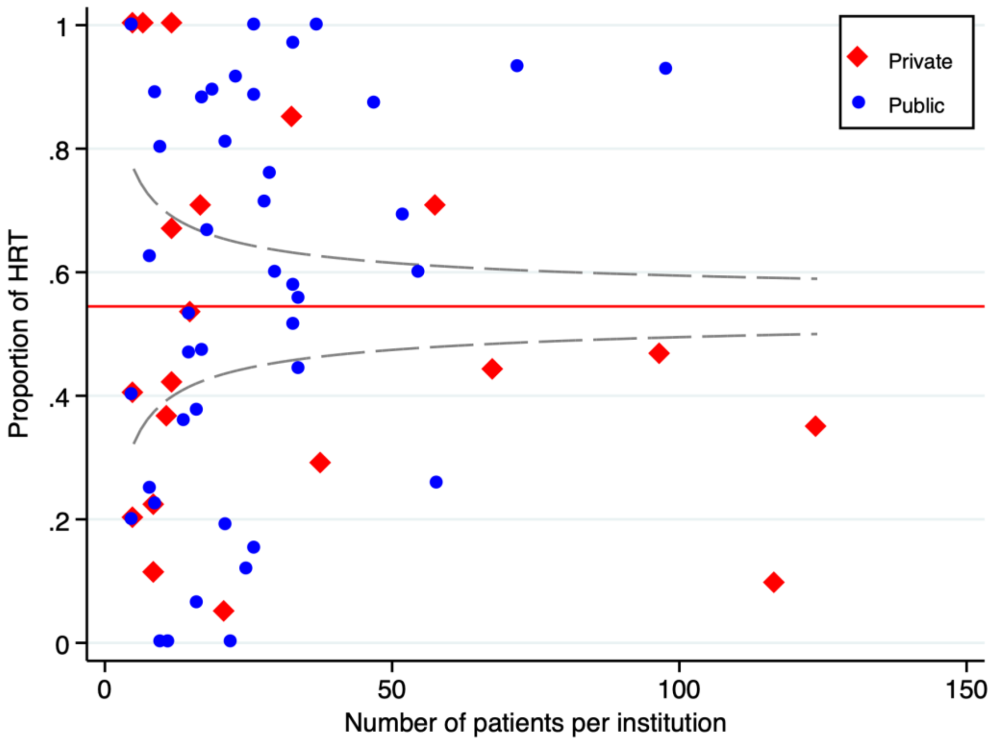

© 2021 Pryor DI et al. *JAMA Network Open*.

eFigure 3. Violin plot of EPIC-26 domain scores for urinary incontinence, urinary irritation/obstruction, bowel function, and sexual function by radiation therapy schedule.

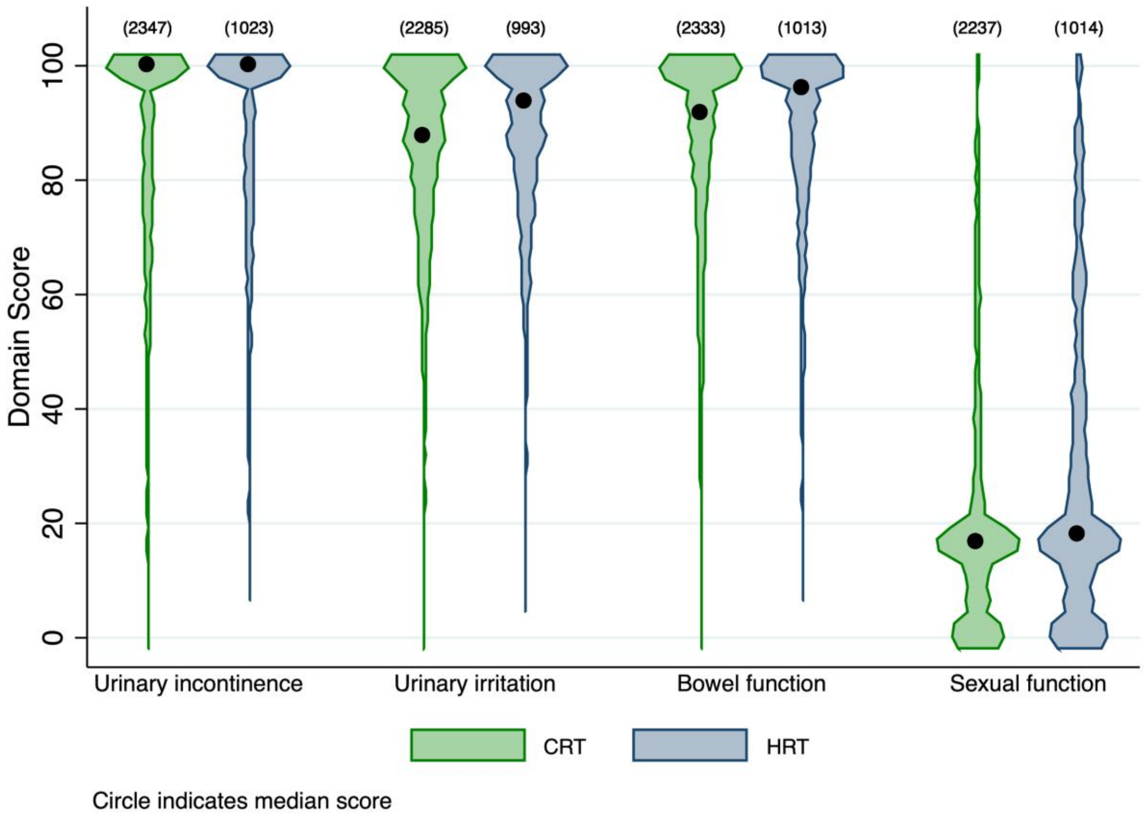

© 2021 Pryor DI et al. *JAMA Network Open*.

eTable 1. Percentage moderately hypofractionated radiation therapy and 95% exact binomial confidence interval (CI) by year and NCCN risk category.

	All patients		Low		Intermediate		High / Very High		Regional (cN1)	
Year	N	% (CI)	N	% (CI)	N	% (CI)	N	% (CI)	N	% (CI)
2016	957	4.8 (3.4 – 6.2)	30	3.3 (0 – 10)	420	6.4 (4.1 – 8.8)	463	3.7 (2.0 – 5.4)	44	2.3 (0 – 6.9)
2017	1447	19 (17 – 21)	45	33 (19 – 48)	667	29 (25 – 32)	645	10 (7.7 – 12)	90	7.8 (2.1 – 13)
2018	1834	29 (27 – 31)	44	48 (32 – 63)	863	43 (39 – 46)	798	17 (14 – 19)	129	7.8 (3.1 – 12)
2019	2130	48 (46 – 50)	49	61 (47 – 75)	961	64 (61 – 67)	930	36 (33 – 40)	190	21 (15 – 27)

© 2021 Pryor DI et al. *JAMA Network Open*.

eTable 2. Percentage moderately hypofractionated radiation therapy and 95% exact binomial confidence interval (CI) by year and jurisdiction for NCCN intermediate risk disease.

	All patients		NSW/ACT		Victoria		Queensland		SA/NT		New Zealand	
Year	N	% (CI)	N	% (CI)	N	% (CI)	N	% (CI)	N	% (CI)	N	% (CI)
2016	420	6.4 (4.1 – 8.8)	101	15 (7.8 – 22)	150	2.7 (0.1 – 5.3)	94	5 (0.7 – 9.9)	45	6.7 (0 – 14)	30	0
2017	667	29 (25 – 32)	212	37 (31 – 44)	208	21 (15 – 26)	117	21 (13 – 28)	77	23 (14 – 33)	53	53 (39 – 67)
2018	863	43 (39 – 46)	223	52 (46 – 59)	243	45 (39 – 51)	186	26 (19 – 32)	105	12 (6.0 – 19)	106	76 (68 – 85)
2019	961	64 (61 – 67)	210	68 (61 – 74)	212	60 (54 – 67)	237	53 (47 – 60)	59	39 (26 – 52)	243	82 (77 – 87)

© 2021 Pryor DI et al. *JAMA Network Open*.

eTable 3. Characteristics of domain specific scores of EPIC-26 in the sample.

Domain	Mean (SD)	% minimum	% maximum	Cronbach's alpha
Urinary Incontinence	86.5 (19.6)	0.1	53.2	0.82
Urinary irritation	86.0 (15.7)	0.1	32.5	0.64
Bowel	86.8 (17.7)	0.1	38.7	0.84
Sexual	24.9 (25.1)	17.1	1.3	0.88

© 2021 Pryor DI et al. *JAMA Network Open*.
